# Preservation of Cichoric Acid Antioxidant Properties Loaded in Heat Treated Lactoferrin Nanoparticles

**DOI:** 10.3390/molecules23102678

**Published:** 2018-10-18

**Authors:** Junyi Li, Caicai Zhao, Liping Wei, Xiang Li, Fuguo Liu, Min Zhang, Xuebo Liu, Yutang Wang

**Affiliations:** 1 Beijing Advanced Innovation Center for Food Nutrition and Human Health, Beijing Technology and Business University, Beijing 100089, China; 18829352098@163.com; 2 College of Food Science and Engineering, Northwest A&F University, Yangling 712100, China; zhaocaicai1994@163.com (C.Z.); 13279492713@163.com (L.W.); 18392143863@163.com (X.L.); fuguo@nwafu.edu.cn (F.L.)

**Keywords:** lactoferrin, cichoric acid, nanoparticles, non-covalent interactions, antioxidant activity

## Abstract

In the current research, a new cichoric acid (CA) encapsulation system was investigated. The optimal condition for the formation of lactoferrin-cichoric acid nanoparticles (LF-CA NPs) was determined by controlling the solution pH, the thermal treatment conditions, and the concentration of CA. Fluorescence indicated that the electrostatic force and the hydrophobic force were the main forces in the formation of LF-CA NPs. LF-CA NPs prepared under different conditions were spherical in shape with smaller particle sizes and good zeta potential demonstrating good colloidal stability. Especially, the prepared particle size of the LF-CA NPs at pH 7 and 95 °C was about 67.20 ± 1.86 nm. The circular dichroism (CD) and the Fourier transform infrared spectroscopy (FTIR) results showed that the combination of LF (lactoferrin) and CA affected the secondary structure of the LF. The differential scanning calorimetry (DSC) results indicated that the addition of CA increased the thermal stability of LF. In vitro antioxidant experiments confirmed the antioxidant capacity of LF-CA NPs was better than CA. CA was successfully encapsulated into LF NPs with high encapsulated efficiency (97.87–99.87%) by high performance liquid chromatography (HPLC). These results showed that LF could be used as the wall material of CA with excellent nature.

## 1. Introduction

Cichoric acid, a natural polyphenolic compound, is an antioxidant in edible plants. CA has been shown to have numerous health-promoting effects, such as scavenge free radicals and antioxidant [1,2,3,4], antiviral [5], and anti-inflammatory properties [6]. But previous studies have shown that CA is unstable in water solution and the gastrointestinal tract, and is prone to enzymatic degradation or oxidation [7,8]. The increase of temperature accelerates the hydrolysis of CA to coffee tartaric acid and caffeic acid [9]. Therefore, how to effectively use CA has great significance for human health.

LF, a natural iron-binding glycoprotein existed in human and mammalian milk extracts, is one of the important inflammatory response regulators, which has a molecular weight of about 80 kDa [10,11]. In addition, LF also has anti-inflammatory and antioxidant effects and is an outstanding food-grade carrier able to encapsulate oxidized nutrients due to its good biocompatibility, biodegradability, low cytotoxicity, and good cost-effectiveness [12,13,14,15]. The delivery system can physically or chemically interact with functional factors and then expressively improve the physical and chemical properties of the functional factors. LF can be used to conjugate with pectin via electrostatic interaction and it can also be used as the carrier of curcumin to improve the water solubility, controlled release, and antioxidant activity of hydrophobic curcumin [16]. Recent studies have also demonstrated that LF can be applied as a carrier of gambogic acid to prepare LF-gambogic acid nanoparticles, moreover, the results of vivo experiments in mice proved that LF-gambogic acid nanoparticles is an effective method for the oral delivery of gambogic acid [17].

Based on the above studies, we found that LF not only has antioxidant capacity, but also can be used in the construction of nanoparticle delivery systems. At present, there is no research on the construction of LF and CA nanoparticles and whether there is synergy between them has not been reported. In the current study, we determined the properties and antioxidant properties of the constructed LF-CA NPs, providing a reference for synergistic antioxidant research between proteins and polyphenols and new ideas for our further research to promote the absorption of CA in vivo.

## 2. Results and Discussion

### 2.1. Result of Fluorescence Measurement of LF

#### 2.1.1. Fluorescence Spectroscopy

**Figure 1:**
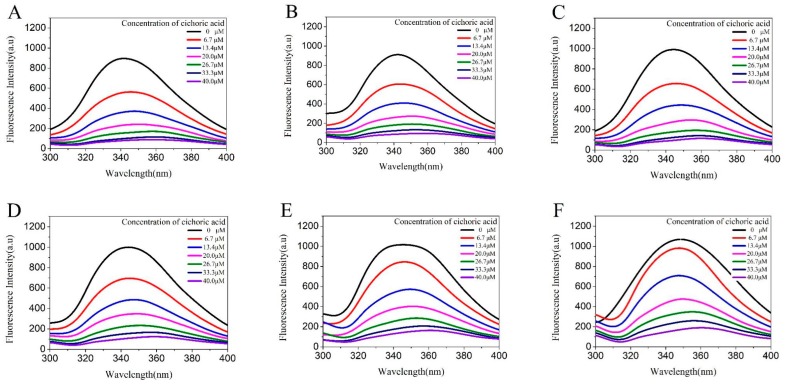
Fluorescence emission spectra of 0.025 M LF at pH 4.0 (curve **A**–**C**) and pH 7.0 (curve **D**–**F**) in the presence of CA with concentrations in the range of 0 to 40 μM upon excitation at 295 nm, and (**A**,**D**) were at 25 °C, (**B**,**E**) were at 70 °C, (**C**,**F**) were at 95 °C.

LF contains three aromatic amino acids: tryptophan, tyrosine, and phenylalanine. The tryptophan fluorescence intensity is the highest among them. The emission maximum of tryptophan in water occurs near 350 nm and is highly dependent upon polarity and/or the local environment. When the tryptophan residues become hydrogen bonds or are exposed to water, the emission shifts to longer wavelengths, that are called red-shifts [18]. The fluorescence intensity of the fluorescent chromophore decreases when molecular interactions, molecular rearrangements, formation of ground state complexes, and collisional quenching occur [19]. As shown in Figure 1, the LF solution has an emission peak around 350 nm, the same as the fluorescence peak of tryptophan, and the fluorescence intensity of the LF solution gradually decreased with the increase of the CA concentration, showing a typical phenomenon of fluorescence quenching, proving that CA interacted with LF and formed a complex with no fluorescence or weak fluorescence. At the same time, with the increase of CA concentration, the maximum emission wavelength of LF was shifted to the long wave direction, indicating that the interaction between LF and CA resulted in the change of the tryptophan environment of LF and the increase of hydrophilicity. In addition, the red-shift of the fluorescence peak indicated that the binding of LF to CA caused changes in its structure, with more side chains exposed to the buffer solution, and tryptophan was also transferred to a more hydrophilic environment [20]. It was demonstrated that the combination of LF and CA caused a change in the structure of LF.

#### 2.1.2. Quenching Type

**Figure 2:**
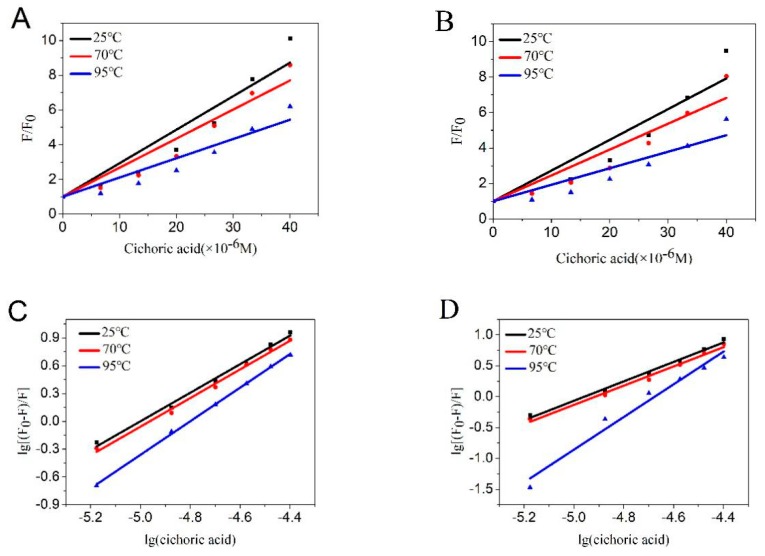
Stern-Volmer plots for LF fluorescence quenching by CA at pH 4 (**A**) and pH 7 (**B**). Double log plots of LF with CA at pH 4 (**C**) and pH 7 (**D**).

After determining the combination of CA and LF, we further analyzed the quenching mechanism. The fluorescence quenching mechanisms of small molecules and proteins are mainly static and dynamic, with major differences in their dependence on temperature. Figure 2A,B are Stern-Volmer plots of the fluorescence quenching of LF in the presence of CA at various temperatures and pH conditions. We can see that the K_sv_ value decreased with the treatment temperature of LF increased, suggesting that the quenching mechanism of the interaction between CA and LF may be static rather than dynamic. The formation of non-fluorescent or weakly fluorescent complexes resulted in fluorescence quenching. When the temperature increased, the complex dissociated and the quenching constant decreased. The K_sv_ value are shown in Table 1.

At 25 °C, 70 °C, and 95 °C, the quenching rate constants of LF were all far greater than those of various quencher pairs. The maximum diffusion collision quenching constant of the macromolecules was 2.0 × 10^10^ mol^−1^ L s^−1^ [21]. This result further demonstrated that the fluorescence quenching of LF by CA is a static quenching due to the formation of a compound with no fluorescence or weak fluorescence.

#### 2.1.3. Binding Constants and Binding Sites

As shown in Figure 2C,D, and Table 2, at pH 4 and pH 7, the binding sites do not change at 25 °C compared to 70 °C, but at 95 °C, the binding sites become larger and approach to 2. It is possible that LF is thermally denatured at 95 °C and further binding occurs with CA.

#### 2.1.4. Thermodynamic Parameters and Types of Action

The non-covalent interaction forces between small molecules and proteins mainly include hydrogen bonding forces, van der Waals forces, hydrophobic interaction forces, and electrostatic interaction forces. The indicators for determining the force mainly include free energy change, transmutation, and entropy change.

When the force is mainly the hydrophobic force, ΔH and ΔS are both greater than zero; when the electrostatic force is the main force, ΔH is less than zero and ΔS is greater than zero; when ΔH and ΔS are both less than zero, the Fan Dehua force or hydrogen bond is the main force. It can be seen from Table 3 that the main force from 25 °C to 70 °C is the electrostatic force and the main force from 25 °C to 95 °C is the hydrophobic force at pH 4 and pH 7.

### 2.2. Characterization of Nanoparticles

#### 2.2.1. UV Light Spectral Scanning

**Figure 3:**
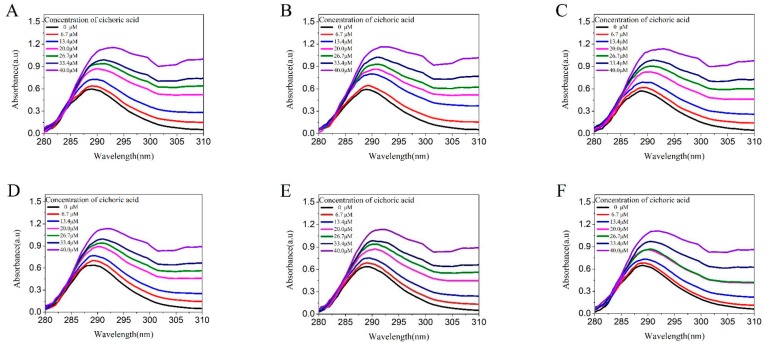
UV–VIS absorption spectra of 25 mM LF at pH 4.0 (curve **A**–**C**) and pH 7.0 (curve **D**–**F**) in the presence of CA with concentrations in the range of 0 to 40 μM upon excitation at 295 nm, and (**A**,**D**) were at 25 °C, the (**B**,**E**) were at 70 °C, and (**C**,**F**) were at 95 °C.

From the results of the UV full-wavelength scan (Figure 3), the absorbance of the solution increased with the increase of the concentration of CA, indicating that the combination of CA and LF caused changes in the protein structure. As shown in Figure 3, the absorption peak of LF gradually increases and red-shift occurs with the increase of CA concentration. Dynamic quenching did not change the absorption spectrum of the protein. The formation of non-fluorescent ground state complexes can change the absorption spectrum of the protein. This result is consistent with the previous fluorescence results [22] and once again demonstrates that the fluorescence quenching of LF is a static quenching [23]. Since CA and LF reached a maximum at the concentration of 40 μM CA, this concentration was used in other experiments thereafter.

#### 2.2.2. EE (%)

**Figure 4:**
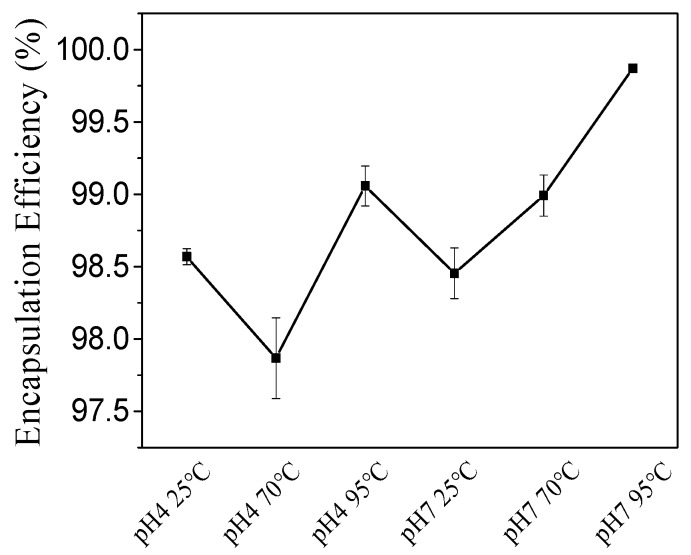
Encapsulation efficiency of CA in LF NPs.

The EE (%) of the LF-CA NPs was successfully determined by HPLC. Then we used the formula to calculate the EE of the LF-CA NPs. We can see from Figure 4 that the combination is at a range of 97.87–99.87%. A high binding rate guaranteed the efficient use of CA and proved the rationality of the method we used and the molar ratio of LF and CA. Such a high EE (%) of the LF-CA NPs could be attributed to the strong electrostatic force and the hydrophobic force. The high EE (%) of the LF-CA NPs along with an optimum particle size and zeta potential may provide an increased advantage under in vivo conditions.

#### 2.2.3. The Particle Size, Zeta-Potential, and PdI

From Table 4, we can see that at the same pH, with the increase of temperature, the change trend of the particle size of LF and LF-CA NPs is roughly the same, but LF-CA NPs formed under the same conditions have smaller particle size. Due to the smaller particle size, we suspected that the addition of CA resulted in a more compact and stable structure of LF. As the pH of the LF solution was lower than the isoelectric point of LF, the surface was positively charged. The heating enhanced the electrostatic repulsion and further inhibited the aggregation of LF NPs. Meanwhile, at elevated temperatures, the auto-oxidation of CA resulted in the formation of free radicals or quinones, and the LF unfolded the native conformation of the protein molecule and exposed the hydrophobic site to the exterior, which can interact with the free radical or hydrazine. Simultaneous formation of covalent disulfide bonds can reduce the size of covalent complexes [24]. At pH 7, the average particle size of LF-CA NPs formed at a heating condition of 95 °C was 67.20 nm (Table 4), much lower than those formed under other conditions. We think that LF may be aggregated and form larger particles due to the new intermolecular interactions under acidic conditions [25]. On this basis, we analyzed the zeta potentials and found that the zeta potentials of LF-CA NPs were smaller than the zeta potential of LF NPs, but they still distributed around 20 mV, indicating that LF-CA NPs have good colloidal stability. From Table 4, the PdI of the solution did not change significantly with the addition of CA. The results show that the NPs formed under this condition not only has the smallest particle size, but also has relatively good colloidal stability. The molecular weight of LF is an average value, and the particle size of pure LF was measured, and there were three peaks that have different average particle sizes except that the PdI value is higher than 0.3. Therefore, we think that the main reason for the higher PdI value in the data is the nature of LF itself.

#### 2.2.4. FTIR

**Figure 5:**
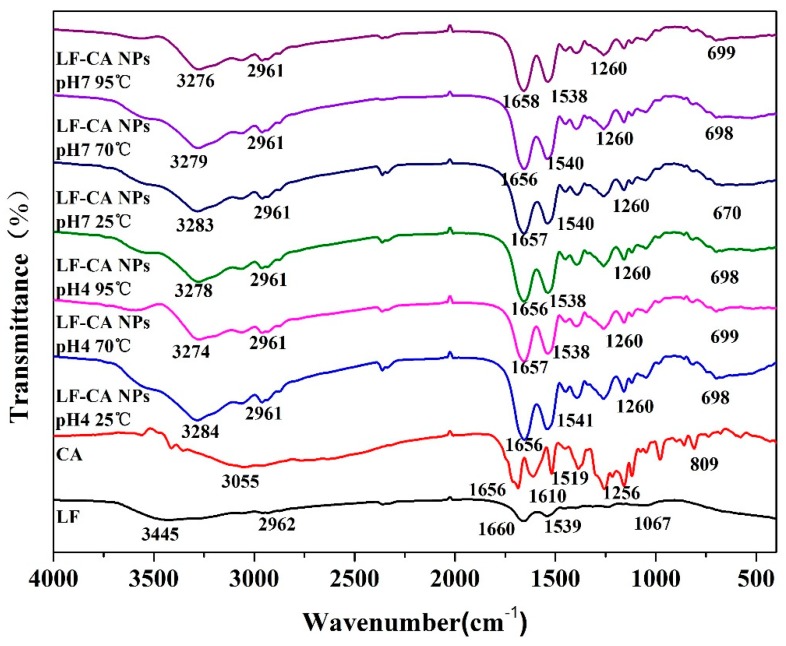
FTIR characteristics of LF-CA NPs, LF NPs, and CA.

Figure 5 shows the FTIR spectra of pure LF and CA and LF-CA NPs prepared using six different conditions. The broad band in 3100–3500 cm^−1^ was attributed to O-H stretching vibration of hydroxyls-bound water. The absorption peak appeared at about 3445 cm^-1^ for LF, and the peak of the LF-CA NPs shifted to about 3270 cm^−1^ [26]. Simultaneously comparing LF-CA NPs with LF, the band of 2800–3000 cm^−1^ was attributed to C–H stretching vibrations, and the peak area between 1500 and 1700 cm^−1^ represented the amide I and amide II groups. Amide I (1600–1700 cm^−1^) was mainly governed by the stretching vibration of the C=O and C=N groups [27], while amid II (1500–1600 cm^−1^) was due to the bending vibration of the N–H groups and the stretching vibrations of the C–N groups. These results suggested that the interaction between LF and CA consisted not only of a hydrogen bond, but also hydrophobic effects due to the exposure of hydrophobic groups. This result echoes the fluorescence results. Additionally, we found new peaks at 698 cm^−1^ and 1260 cm^−1^ for LF-CA NPs. New detected peaks suggested that LF and CA were not physically attached to each other but formed a binary biopolymer complex with non-covalent interaction [28], which might result from bonding between the stretching vibrations of C=C and C=O [29].

#### 2.2.5. CD

**Figure 6:**
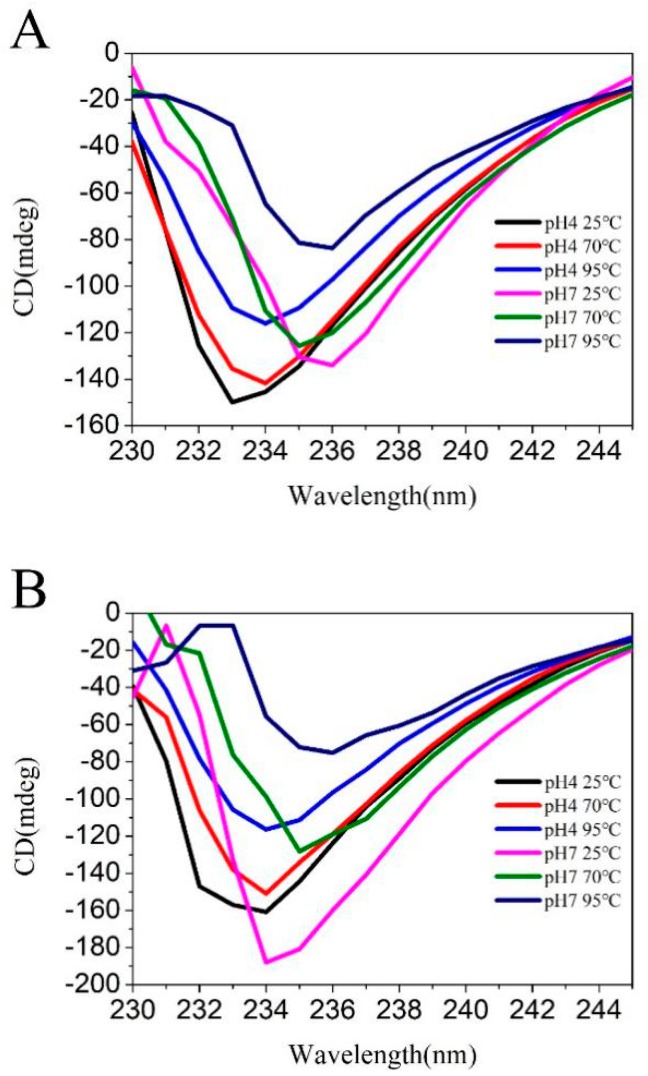
Far-UV CD spectra of LF NPs (**A**) and LF-CA NPs (**B**).

The far-UV region of CD is commonly used to detect the secondary structure of proteins. In this study, the secondary structure changes of LF after binding to CA were determined by CD spectroscopy. As can be seen from Figure 6, with the increase of temperature, there is no significant deviation in the LF chromatographic peak, but the intensity of the peak has changed by varying degrees, and the treatment of pH results in the intensity of the chromatographic peak and the peak of the solution. Both temperature and pH resulted in changes in the secondary structure of the protein. The change trend of the peak intensity was more obvious in the solution containing CA than in the pure LF solution. To further demonstrate the changes in protein structure, we used CDNN software to analyze the results of CD to fit the secondary structure of the protein. The results are shown in Table 5. From Table 5, the α-helical content of the LF solution without CA changes significantly at 70 °C, but the change of the α-helix content becomes smaller after CA is added under the same conditions, demonstrating that LF-CA NPs have a more stable structure. At the same time, the LF-CA NPs have a relatively lower α-helix content compared to the LF solution, and the β-sheet content and random coil content are increased. Compared with α-helix, β-sheet is a more ordered and compact structure with higher relative content and stability. Therefore, the LF-CA NPs structure is more stable than LF. In addition, we compared the samples prepared under six different conditions, and it can be clearly seen that the α-helix in the sample at pH 7 and 95 °C is the lowest, 18.9% for the LF solution, 18.0% for the LF-CA NPs, and the β-sheet and β-translocation levels were also higher than in other samples. Therefore, we believe that the NPs prepared at pH 7 and 95 °C have a more stable structure, and this result is consistent with results of the particle size and zeta potential.

#### 2.2.6. DSC

**Figure 7:**
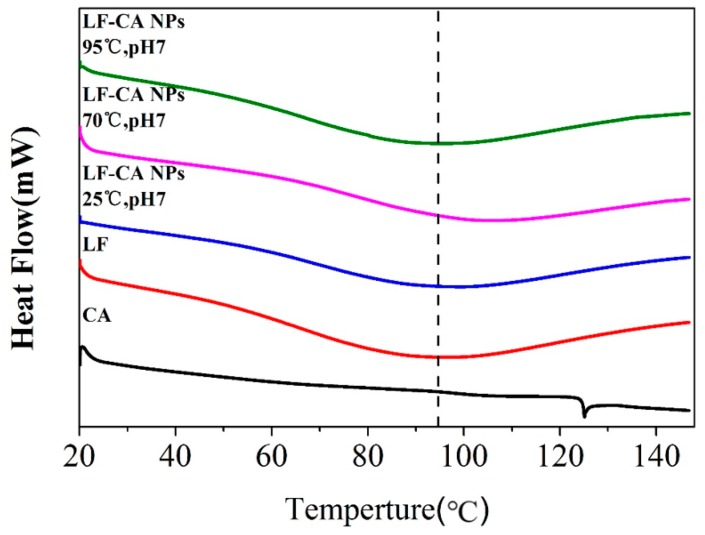
DSC of LF-CA NPs, LF NPs, and CA.

As an effective thermal analysis method, we performed DSC on the thermal stability of LF and LF-CA NPs. From Figure 7, the endothermic peak of the pure LF sample is 95.80 °C, the denaturation temperature of CA is 125.04 °C, and this peak disappears on the LF-CA NPs spectrum, demonstrating the combination of LF and CA. At the same time, the denaturation temperatures of the LF-CA NPs prepared under six conditions are increased by 4–10 °C. This result indicated that the addition of CA increases the thermal stability of LF and demonstrated that the complexation of proteins and polyphenols formed under non-enzymatic oxidative conditions is higher than that of single proteins [30]. This result corresponded to the particle size results that the addition of CA caused the LF to form a smaller and denser structure, resulting in an increase in the thermal stability of LF.

#### 2.2.7. Morphological Analysis

**Figure 8:**
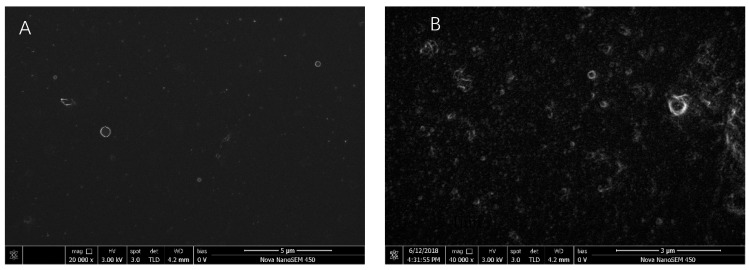
SEM images of LF NPs (**A**) and LF-CA NPs (**B**).

To further explore the microstructure changes of LF and LF-CA NPs, we employed SEM. The spherical morphology and the nanometer size of the prepared NPs were confirmed. As shown in Figure 8, LF-CA NPs are spherical and have a smooth surface and a particle size of about 300–500 nm. In addition, when we observed LF-CA NPs with a heating temperature of 95 °C and pH 7, most of the NPs were at a range of 60–70 nm. These data supported our particle size measurement results.

### 2.3. In Vitro Antioxidant Activities

#### 2.3.1. DPPH Radical Scavenging Activity

**Figure 9:**
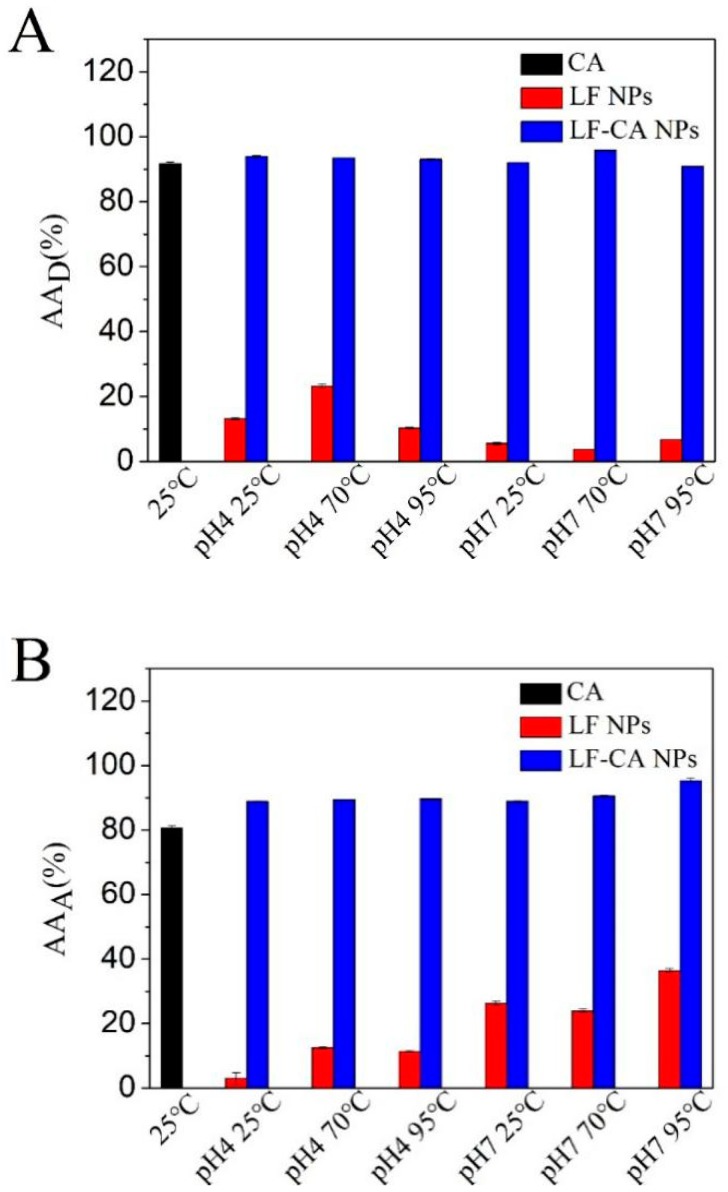
DPPH radical scavenging activity, and Hydroxyl radical scavenging activity of CA, LF NPs, and LF-CA NPs (**A**: DPPH radical scavenging activity; **B**: Hydroxyl radical scavenging activity).

The degree of discoloration of the DPPH solution is taken as the index of the free radical scavenging potential. From Figure 9A, we can see the averaged DPPH inhibition % for the solution. At 20 μmol/L, the scavenging activities of LF-CA NPs are at a range of 90.87–96.55%. Additionally, there was no significant difference with the scavenging activities of CA (91.70%). At 10 μmol/L of LF, the scavenging activities was at a range of 3.75–13.16%. Thus, the combination of LF and CA did not decrease the radical scavenging activity of CA.

#### 2.3.2. ABTS Radical Scavenging Experiment

The same trend was found in the ABTS radical scavenging activities. From Figure 9B, we can see the averaged ABTS inhibition % for the solution. At 20 μmol/L, the ABTS radical scavenging activities of the LF-CA NPs were at a range of 88.83–95.30%, which was increased more than that of CA (80.52%). At 10 μmol/L of LF, the ABTS radical scavenging activities was at a range of 3.04–36.46%. These antioxidant results showed that when LF and CA were applied together, the antioxidant ability of LF-CA NPs was increased with no significant difference. Since the standard error is too small, it is not obvious in the Figure 9. Since the combination of most macromolecular substances will adversely affect the antioxidant properties of the functional factors themselves [31]. Therefore, we believe that the phenomenon that the oxidation resistance of CA has not increased is an acceptable result.

## 3. Materials and Methods

### 3.1. Materials and Chemicals

LF was purchased from PROCHIN international Trading Co., Ltd. (Shanghai, China). CA was purchased from Sigma-Aldrich Co. (St. Louis, MO, USA). All other analytical grade chemicals and reagents were obtained from Sino Pharm Chemical Reagent Co., Ltd. (Shanghai, China).

### 3.2. Preparation of LF-CA Nanoparticles

In brief, 0.4 g LF was added to 100 mL ultrapure water, and the mixture was stirred magnetically for two hours to fully dissolve the LF; 0.1 mM HCl and NaOH was gradually added to adjust the pH to 4 and 7 while the solution was constantly stirred. Then we heated the LF mixture and bathed it in water at 25 °C, 70 °C, and 95 °C for 20 min for further use. A total of 23.7 mg CA was added to 100 mL ultrapure water, which was stirred magnetically for 2 h. Then, we added different concentrations (0.0, 6.7, 13.4, 20.0, 26.7, 33.3, and 40.0 μM) of CA to the prepared LF solution and quickly cooled the solutions. The samples were then vortexed for 2 min to allow them to fully react. Finally, we lyophilized the solutions and stored them at −20 °C. However, it is worth noting that the samples used, except for FTIR and DSC needed to be newly configured to prevent changes in the properties of LF-CA NPs during storage.

### 3.3. Fluorescence Spectroscopy

A constant LF concentration (10 μM) in the presence of 0, 6.7, 13.4, 20.0, 26.7, 33.3, and 40.0 μM CA was measured by the fluorescence spectroscopy using a Hitachi F4500 fluorescence spectrometer (Tokyo, Japan). Emission spectra were measured from 300 to 500 nm at an excitation wavelength of 295 nm. The spectral resolutions of both excitation and emission were 5 nm. In this study, the fluorescence spectra of the controls were subtracted from the respective spectra of the samples to offset any contribution that was due to the Raman peak and other scattering artifacts.

The K_sv_ value was calculated according to the following equation:

$$\;F_{0}/F=1+K_{\mathrm{SV}}\left[Q\right]=1+K_{q}\tau_{0}\left[Q\right]\;$$

where F_0_ and F, respectively, represent the fluorescence intensity in the absence of CA and the concentration of CA is [Q], K_q_ is the quench rate constant, K_sv_ is the dynamic quenching constant, and τ_0_ is the average life span of biological macromolecules in the absence of quenchers, approximately 10^−8^ s.

The calculation of the binding constants and binding sites is based on the double logarithmic formula:

$$\;\mathrm{lg}\left[F_{0}-F/F\right]={\mathrm{lgK}}_{a}+\mathrm{nlg}\left[Q\right]\;$$

where K_a_ is the binding constant and n is the number of binding sites.

Free energy change, transmutation, and entropy change were calculated according to the following formulas:

$$\;{\mathrm{InK}}_{a2}/K_{a1}=1/R\left[1/T_{1}-1/T_{2}\right]\Delta H\;$$

$$\;\Delta G=\Delta H-T\Delta S=-{\mathrm{RTInK}}_{a}\;$$

where k_a1_ and k_a2_ are the binding constants under temperature T_1_ and T_2_, R is the gas constant, ∆G, ∆H and ∆S are, respectively, free energy changes, enthalpy changes, and entropy changes.

### 3.4. UV Light Spectral Scanning

We performed a 200–450 nm full-wavelength scan of samples with UV-2550 (Shimadzu, Japan) to observe the change in absorbance of the sample while finding the maximum absorption wavelength of the sample. Ultrapure water was used as blanks.

### 3.5. Encapsulation Efficiency (EE)

The EE (%) of the LF-CA NPs was determined according to a reported method [32] by the HPLC system comprising a binary LC-20C D pump, a SIL-20AHT autosampler, and a column oven (Shimadzu, Tokyo, Japan). To efficiently separated free CA, we used the Amicon Ultra-4 centrifugal filter devices (Millipore Co., Billerica, MA, USA) made up of a centrifuge tube and a filter unit with a low-binding Ultracel membrane (MWCO 3000) [33]. The 1 mL samples were dissolved in 2 mL methanol containing 2 mL 0.5% phosphoric acid and then sonicated for 15 min. Finally, needle syringes and 0.45 μM microporous membranes were used to filter the samples. The mobile phase (water containing 0.2% phosphoric acid: acetonitrile at a ratio of 75:25 *v*/*v*) was delivered with a flow rate of 1.0 mL/min at 35 °C, and separation was performed using an Agilent Eclipse column XDB-C18 (5 mm, 4.6 × 150 mm) with a sample injection volume of 20 μL. The wavelength for detection of CA was 327 nm. Results (EE (%)) were calculated using the following equation:

 EE (%)=[1−(amount of CA in ultrafiltrate /total amount of CA)]×100 

### 3.6. Particle Size, Zeta-Potential and Polydispersity Index

The average particle size, zeta potential, and polydispersity index (PdI) of different formulations were evaluated with Nano-ZEN 360 (Malvern Instruments, Worcestershire, UK) by dynamic light scattering. The refraction index used here was 1.330. The particle size data was reported as a cumulative mean diameter (size, nm). At the same time, we used this instrument to determine the zeta potential, particle size, and PdI of different solutions. All samples were measured in triplicate at 25 °C.

### 3.7. FTIR

At the beginning, we mixed the sample powder with KBr in a 1:100 ratio. Then, characterizations of pure LF, CA, and LF-CA were scanned with FTIR (Vertex 70, Bruker, Germany) in the wave number range of 400–4000 cm^−1^ with KBr pellets and referenced against that of air. All spectra were collected at 4 cm^−1^ resolution. A background spectrum was obtained for each sample. The powder sample was placed on the center of the crystal surface. Sixteen scans were taken for the background and the sample, and the spectral collection of each sample was measured three times under the same conditions [34].

### 3.8. CD

The secondary structures of proteins in covalent complexes were also studied by CD spectropolarimeter (Jasco J-815, Chirascan, Japan) between 180 and 250 nm with an interval of 1 nm at 25 °C. The measurement was carried out at room temperature under a continuous nitrogen atmosphere with a scan rate of 100 nm/min, a bandwidth of 2.0 nm and a path length of 1 mm. We measured the freshly prepared samples to determine the secondary structures at 0.8 mg/mL. The ultrapure water was used as the blank for all samples. Three scans were averaged to obtain one spectrum. The secondary structure compositions were calculated from far-UV CD spectra data using CNDD software (version 2.1, Applied Photophysics Ltd., Leatherhead, UK).

### 3.9. DSC

DSC was carried out using the DSC Q2000 instrument (TA Instruments, New Castle, DE, USA). We weighed 3 mg samples placed in an aluminum sample box, sealed the box, and then heated the samples from 20 to 150 °C at 10 °C/min and sealed an empty aluminum box as a blank control. The flow rate of dry nitrogen was 50 mL/min [35].

### 3.10. Scanning Electron Microscope (SEM)

We used freeze-dried powder samples and newly-configured liquid samples to observe changes in the morphology structures of the NPs under scanning electron microscopy (Nova Nano SEM-450, FEI NanoPorts, Hillsboro, OR, USA). The particle size and shape were also observed. Freeze-dried NPs powders were added and adhered onto conductive carbon tape. The liquid sample was placed on a silicon wafer and then dried using a lamp. Gold particles were deposited onto the powders and the silicon wafer with a sputter coater under vacuum to avoid the charging effect prior to the observation. An electron accelerating voltage of 3.00 kV was used. Representative images were reported.

### 3.11. In Vitro Antioxidant Activities

The in vitro antioxidant activities of LF, CA, and LF-CA NPs were evaluated by 2,2-diphenyl-1-picryl-hydrazil (DPPH) radical scavenging activity and hydroxyl radical scavenging activity. DPPH radical scavenging activity was determined according to a method that has been reported previously [36]. 1 mL of sample was added to 2 mL of 0.1 mM DPPH dissolved in ethanol and agitated for 10 s, then kept for 30 min at room temperature. The concentration of CA as LF-CA NPs formulation and free is 20 μM. And the concentration of LF as LF-CA NPs formulation and free is 10 μM. The absorbance was read at 517 nm. The radical scavenging activity (AA_D_) was calculated according to the following equation:

$$\;{\mathrm{AA}}_{D}\left(\%\right)=100\times \left(A_{C}-\left(A_{S}-A_{B}\right)\right)/A_{C}\;$$

where A_C_ represents the absorbance of the control, A_S_ the absorbance of the sample after LF-CA NPs were added, and A_B_ the absorbance of LF-CA NPs alone.

The 2,2′-azino-bis(3-ethylbenzothiazoline-6-sulphonic acid) (ABTS) radical scavenging experiment was modified in a simple way according to the previous method [37]. The 7 mmol/L ABTS solution and 7.35 mmol/L K_2_S_2_O_8_ solution were mixed according to the proportion of 2:1, and the ABTS^+^ reserve solution was formed by reaction with 12–16 h at 25 °C. ABTS reserve liquid should be diluted to meet the absorbance of 0.70 + 0.02 at 734 nm. The LF, CA, and LF-CA NPs were added to the diluted ABTS solution (volume ratio 1:3), oscillated 10 s. After 5 min, it was measured under 734 nm. Results (AA_H_) were calculated using the following equation:

$$\;{\mathrm{AA}}_{A}\;\left(\%\right)=100\times (\left(A_{C}-\left(A_{S}-A_{B}\right)\right)/A_{C})\;$$

where A_C_ represents the absorbance of the control, A_S_ the absorbance of the sample after LF-CA NPs were added, and A_B_ the absorbance of LF-CA NPs alone.

### 3.12. Statistical Analysis

All measurements were performed at least three times and were reported as mean ± standard error. The data were analyzed by analysis of variance (ANOVA) using the SPSS 17.0 package (IBM, New York, NY, USA). Duncan’s multiple range test was used to determine the significant differences of the mean values (*p* < 0.05).

## 4. Conclusions

In this research, LF-CA NPs were constructed successfully. The combination of LF-CA was proved by the use of fluorescence spectroscopy, the main forces were further defined as the electrostatic force and the hydrophobic force. Compared with pure LF, the LF-CA NPs prepared at pH 7 and 95 °C had higher thermal stability and antioxidant activity, with a smaller average particle size of 67.20 nm, and a zeta potential of 20–30 mV at a high binding rate. Further studies will be focused on determining the biological activity of the complex NPs, such as cellular uptaking study and antioxidant capacity in vivo. We are aimed at the application of LF-CA NPs to oral delivery of CA, reducing the degradation rate of CA in the stomach and improving intestinal absorption.

## Figures and Tables

**Table 1:** Stern-Volmer quenching constants of CA and LF at pH 4 and pH 7.

Temperature (°C)	pH	K_sv_ (×10^5^ L/mol)	K_q_ (×10^13^ L mol^−1^·S^−1^)	r
25	4	1.702	1.702	0.955
70	4	1.531	1.531	0.958
95	4	0.983	0.904	0.951
25	7	1.475	1.475	0.957
70	7	1.257	1.257	0.955
95	7	0.783	0.783	0.944

**Table 2:** The binding constants and binding sites of LF and CA at pH 4 and pH 7.

Temperature (°C)	pH	K_a_ (L/mol)	n	r
25	4	4.94 × 10^7^	1.539	0.996
70	4	4.55 × 10^7^	1.543	0.997
95	4	4.92 × 10^8^	1.811	1.000
25	7	6.14 × 10^7^	1.571	0.996
70	7	4.30 × 10^7^	1.553	0.995
95	7	1.88 × 10^10^	2.178	0.997

**Table 3:** The thermodynamic parameters for the binding of LF and CA at pH 4 and pH 7.

Temperature (°C)	pH	△G (kJ·mol^−1^)	△S (J·mol^−1^·k^−1^)	△H (KJ·mol^−1^)
95	4	−61.23	247.74	29.93
70	4	−50.29	142.10	−1.55
25	4	−43.89		
95	7	−72.38	399.27	74.55
70	7	−50.12	126.54	−6.72
25	7	−44.43		

**Table 4:** Composition and physiochemical characteristics of LF NPs and LF-CA NPs. Different letters (a–e) in the same column indicated significant differences (*p* < 0.05).

Temperature (°C)	pH	LF NPs			LF-CA NPs		
		Particle Size (nm)	PdI	Zeta Potential (mV)	Particle Size (nm)	PdI	Zeta Potential (mV)
25 °C	4	492.63 ± 16.24a	0.49 ± 0.02a	22.33 ± 0.96b	473.70 ± 12.38a	0.45 ± 0.04b	19.87 ± 0.61b
70 °C		543.40 ± 29.00a	0.55 ± 0.03a	26.23 ± 2.55a	314.50 ± 6.70c	0.69 ± 0.05a	27.97 ± 1.30a
95 °C		485.30 ± 10.94a	0.45 ± 0.01b	26.73 ± 0.77a	437.23 ± 10.58b	0.48 ± 0.03b	25.47 ± 0.05a
25 °C	7	346.63 ± 19.32b	0.42 ± 0.05b	21.70 ± 0.16b	344.47 ± 16.72c	0.38 ± 0.01c	15.98 ± 0.24c
70 °C		106.37 ± 8.02c	0.52 ± 0.03a	25.83 ± 0.90a	86.69 ± 6.94d	0.51 ± 0.11ab	16.63 ± 0.66c
95 °C		75.75 ± 2.16d	0.45 ± 0.04b	27.53 ± 0.68a	67.20 ± 1.86e	0.47 ± 0.02b	22.03 ± 1.18b

**Table 5:** Secondary structure elements of LF and LF in LF-CA NPs formed at different conditions.

Temperature (°C)	pH	LF NPs				LF-CA NPs			
		α-Helix (%)	β-Sheet (%)	β-Turns (%)	Random Coil (%)	α-Helix (%)	β-Sheet (%)	β-Turns (%)	Random Coil (%)
25	4	19.30 ± 0.10	41.30 ± 0.20	19.63 ± 0.03	45.85 ± 0.05	20.30 ± 0.90	37.95 ± 3.45	19.20 ± 0.50	47.00 ± 0.60
70		19.60 ± 0.50	40.00 ± 2.35	19.40 ± 0.40	46.20 ± 0.70	19.35 ± 1.75	40.25 ± 3.45	19.25 ± 0.05	47.40 ± 4.20
95		19.20 ± 0.75	41.90 ± 2.05	19.70 ± 0.15	47.00 ± 1.15	18.60 ± 0.60	42.15 ± 1.75	19.40 ± 0.10	49.15 ± 0.25
25	7	19.80 ± 0.05	40.20 ± 0.25	20.00 ± 0.10	46.10 ± 0.35	20.15 ± 0.50	36.20 ± 0.50	18.65 ± 0.50	50.40 ± 0.60
70		21.50 ± 0.60	35.30 ± 2.45	19.10 ± 0.45	44.30 ± 1.25	19.50 ± 0.10	39.70 ± 0.50	19.25 ± 0.05	48.55 ± 0.55
95		18.90 ± 0.40	43.30 ± 1.25	19.90 ± 0.10	46.60 ± 0.45	17.95 ± 1.25	47.80 ± 4.90	20.50 ± 0.40	46.35 ± 1.65
